# SPARC: Structural properties associated with residue constraints

**DOI:** 10.1016/j.csbj.2022.04.005

**Published:** 2022-04-07

**Authors:** Andrew F. Neuwald, Hui Yang, B. Tracy Nixon

**Affiliations:** a Institute for Genome Sciences and Department of Biochemistry & Molecular Biology, University of Maryland School of Medicine, 670 W. Baltimore Steet, Baltimore, MD 21201, USA; b Department of Biology. Penn State University, 304A Frear South Building, University Park, PA 16802; c Department of Biochemistry and Molecular Biology, 335 Frear South Building, University Park, PA 16802, USA

**Keywords:** Bayesian, Markov chain Monte Carlo, Direct coupling analysis, Computer algorithms

## Abstract

SPARC facilitates the generation of plausible hypotheses regarding underlying biochemical mechanisms by structurally characterizing protein sequence constraints. Such constraints appear as residues co-conserved in functionally related subgroups, as subtle pairwise correlations (i.e., direct couplings), and as correlations among these sequence features or with structural features. SPARC performs three types of analyses. First, based on pairwise sequence correlations, it estimates the biological relevance of alternative conformations and of homomeric contacts, as illustrated here for death domains. Second, it estimates the statistical significance of the correspondence between directly coupled residue pairs and interactions at heterodimeric interfaces. Third, given molecular dynamics simulated structures, it characterizes interactions among constrained residues or between such residues and ligands that: (a) are stably maintained during the simulation; (b) undergo correlated formation and/or disruption of interactions with other constrained residues; or (c) switch between alternative interactions. We illustrate this for two homohexameric complexes: the bacterial enhancer binding protein (bEBP) NtrC1, which activates transcription by remodeling RNA polymerase (RNAP) containing σ^54^, and for DnaB helicase, which opens DNA at the bacterial replication fork. Based on the NtrC1 analysis, we hypothesize possible mechanisms for inhibiting ATP hydrolysis until ADP is released from an adjacent subunit and for coupling ATP hydrolysis to restructuring of σ^54^ binding loops. Based on the DnaB analysis, we hypothesize that DnaB ‘grabs’ ssDNA by flipping every fourth base and inserting it into cavities between subunits and that flipping of a DnaB-specific glutamine residue triggers ATP hydrolysis.

## 1 Introduction

A major goal of modern biology is to identify the molecular determinants and mechanisms underlying protein function. One approach to achieving this goal is to characterize the sequence and structural constraints shared by evolutionarily related proteins with similar functions. Such constraints often appear as conserved residues within an alignment of functionally related proteins from phylogenetically diverse organisms or as sequence covariance in multiple alignments.

Bayesian Partitioning with Pattern Selection (BPPS) [1], [2] identifies patterns of conserved residues arising through functional divergence. It relies on the observation that phylogenetically distant, yet functionally related proteins often conserve non-catalytic residues that evolutionarily related, but functionally divergent proteins do not. This occurs as superfamily members diverge into subgroups, each adapting the superfamily’s structural core to fill a functional niche. Often a subgroup G diverges further into smaller subgroups, each conserving residues constrained by G’s function as well as other residues constrained by more specialized functions. Repeated rounds of such divergence have led to hierarchically arranged subgroups, each of which conserves distinctive residues at specific positions. BPPS identifies and characterizes these subgroups by partitioning a multiple sequence alignment (MSA) into a hierarchically nested series of MSAs, termed a hiMSA, based on correlated residue patterns distinctive of each subgroup. Using Markov chain Monte Carlo (MCMC) sampling, BPPS searches among alternative hiMSAs for one corresponding to a statistical model that is most likely to have generated the aligned sequences. During sampling each protein is assigned to a functionally divergent subgroup based on conserved residues distinguishing that subgroup from other, closely related subgroups. Hence, this process reveals likely sequence and structural determinants of protein function at each level (i.e., superfamily, family, subfamily, etc.) of a hierarchy.

Presumably, such subgroup-specific residues are functionally associated. Consequently, they may mutually interact more often than one would expect by chance. We developed the SIPRIS (Structurally Interacting Pattern Residues’ Inferred Significance) program [3] to estimate the statistical significance of 3D interaction networks involving BPPS-defined pattern residues. For each subgroup of interest, SIPRIS identifies the statistically most significant correspondence between pattern residues and a structurally defined residue cluster. (SIPRIS applies a statistical procedure, termed Initial Cluster Analysis (ICA) [4], to estimate the probability of the observe correspondence between two sets of variables by chance alone.) Pattern residues and the structural clusters are defined in the absence of structural and sequence information, respectively. Hence, BPPS-SIPRIS analyses often elucidate (statistical) sequence/structural properties that conventional computational and experimental approaches fail to detect.

Direct Coupling Analysis (DCA) [5], [6], [7], [8], [9], [10], [11], [12], [13] predicts structurally interacting residue pairs based on sequence covariance within an MSA. The rationale behind DCA is that, over evolutionary time, mutations at a given residue position are compensated for by mutations at interacting positions to thereby maintain structural integrity. DCA avoids the confounding effect of indirect correlations due, for example, to two residues both interacting with a third residue, but not with each other. Because a family of proteins need not conserve the residues participating in such directly coupled (DC) pairs, such correlations may be quite subtle. To assess the statistical significance of the correspondence between high DC-scoring residue pairs and 3D contacts, we developed the STARC (Statistical Tool for Analysis of Residue Couplings) [14] program, which assumes that higher DC-scores should be preferentially associated with closer structural distances. STARC takes as input a list of DC scores (i.e., average product corrected Frobenius norms) for pairs of column positions in an MSA and a set of protein structural coordinates corresponding to one of the aligned sequences. STARC applies ICA to estimate the probability, by chance, of the observed correspondence between the highest DC-scoring pairs and structural contacts. STARC returns an estimated *p*-value expressed as a score *S* = –log_10_(*p*). Viewing direct couplings as functionally imposed, *S* measures the degree to which a 3D structure is in a functionally relevant conformation.

We incorporated BPPS, DCA, SIPRIS, STARC and other procedures into a single program, DARC (Deep Analysis of Residue Constraints) [15], which also aids the visualization of these various constraints, characterizes how they correlate with each other and with structure, and estimates statistical significance. To help identify determinants of protein functional specificity, DARC highlights within sequence alignments and available structures those residues subject to the strongest of each type of constraint. DARC also identifies statistically significant direct couplings across homomeric interfaces, though not across heteromeric interfaces.

Interpreting the biological relevance of such constraints requires further characterization of DARC-defined residues within alternative ligand-bound states and conformations observed among various crystal and cryo-EM structures and (since protein structures are not static) among molecular dynamics (MD) simulated structures. To accomplish this, here we introduce SPARC (Structural Properties Associated with Residue Constraints). SPARC identifies: (i) those protein structures whose highest DC-scoring pairs best correspond to 3D structural interactions with a view to examining the most relevant protein structures in greater detail; (ii) direct couplings between residues at both homomeric and heteromeric interfaces within protein complexes; (iii) the formation of various hydrogen bond interactions and 3D clusters involving top DC-scoring residue pairs and BPPS-defined pattern residues within a time series of MD simulated structures; and (iv) correlated formation or dissociation of interactions involving one or two pairs of residues within MD simulated structures. We illustrate SPARC by applying it to death domains [16], [17], to various enzyme and regulatory heterodimeric complexes, and to two types of homomeric ATPase complexes [18]: bacterial enhancer-binding proteins (bEBPs) and DnaB helicases.

## 2 Results

### 2.1 The SPARC program

SPARC runs in eleven different modes. Two modes (‘*rank*’ and ‘*hetmer*’) are primarily applied to experimentally based structures and these, along with a third mode (‘*simul*’) for simulated structures, compute STARC *S*-scores based on input structures and on DC-scores obtained from a corresponding MSA or, for the *hetmer* mode, from two MSAs. The *rank* mode computes S-scores for each protein subunit of known structure in the MSA, whereas the *hetmer* mode computes *S*-scores across heteromeric subunits—where each DC-coupled pair includes a residue from one subunit and another from a second subunit. The *simul* mode computes *S*-scores over a time series of MD simulated structures of a given protein; this assesses whether the estimated functional relevance of a structure increases, decreases, or stays about the same over time.

The seven remaining analysis modes are also applied to time course MD simulations. These are:

• *sc2sc*: investigates sidechain-to-sidechain hydrogen bonds.
• *sc2bb*: investigates sidechain-to-backbone hydrogen bonds.
• *sc2sb*: runs both sc2sc and sc2bb modes concurrently.
• *dist*: reports residue-to-residue or residue-to-ligand distances.
• *correl*: finds interacting residue pairs that form or dissociate in a correlated manner.
• *bb2bb*: finds backbone-to-backbone interactions.
• *sipris*: finds the most significant SIPRIS clusters.

For all seven modes, SPARC automatically identifies structural interactions or 3D clusters involving BPPS-defined residues and top DC-scoring residue pairs, such as we further describe and illustrate in the following sections. An additional mode, *vsi2pml*, is used to create PyMOL scripts for visualizing residue interactions within MD simulated structures.

### 2.2 Ranking protein structures by *S*-score

In the *rank* mode, SPARC first computes DCA scores by applying the CCMpred [19] algorithm to an input MSA of related proteins. Next, it searches for aligned protein sequences that correspond to one or more structural coordinate files. (Proteins of known structure must be labeled with NCBI pdbaa formatted identifiers, e.g., as 3M0E_A.) Finally, as a measure of biological relevance, it ranks protein structures based on STARC *S*-scores. (Paths to corresponding 3D coordinates, ideally with modeled hydrogen atoms, must also be provided as input. Hydrogen atoms may be modeled using the Reduce program [20] or the PyMOL h_add command.) SPARC also computes the change in *S*-score (Δ*S*) upon inclusion of homomeric interactions, when present; values of Δ*S* ≥ ∼3.0 suggest that some residue pairs are directly coupled due to interactions between identical subunits. *S* and Δ*S* scores can help identify the biologically most relevant proteins structures for further analysis. In addition to using a superfamily MSA to compute SPARC *S*-scores, it is often more informative to apply it to a subgroup-specific MSA, which may be obtained using DARC, to characterize subgroup-specific DC constraints. Hence, our approach is to first use SPARC to search the superfamily MSA for high *S*- and Δ*S*-scoring proteins, each of which may then be used as a query for DARC to define a subgroup MSA for a second SPARC analysis.

**Fig. 1:**
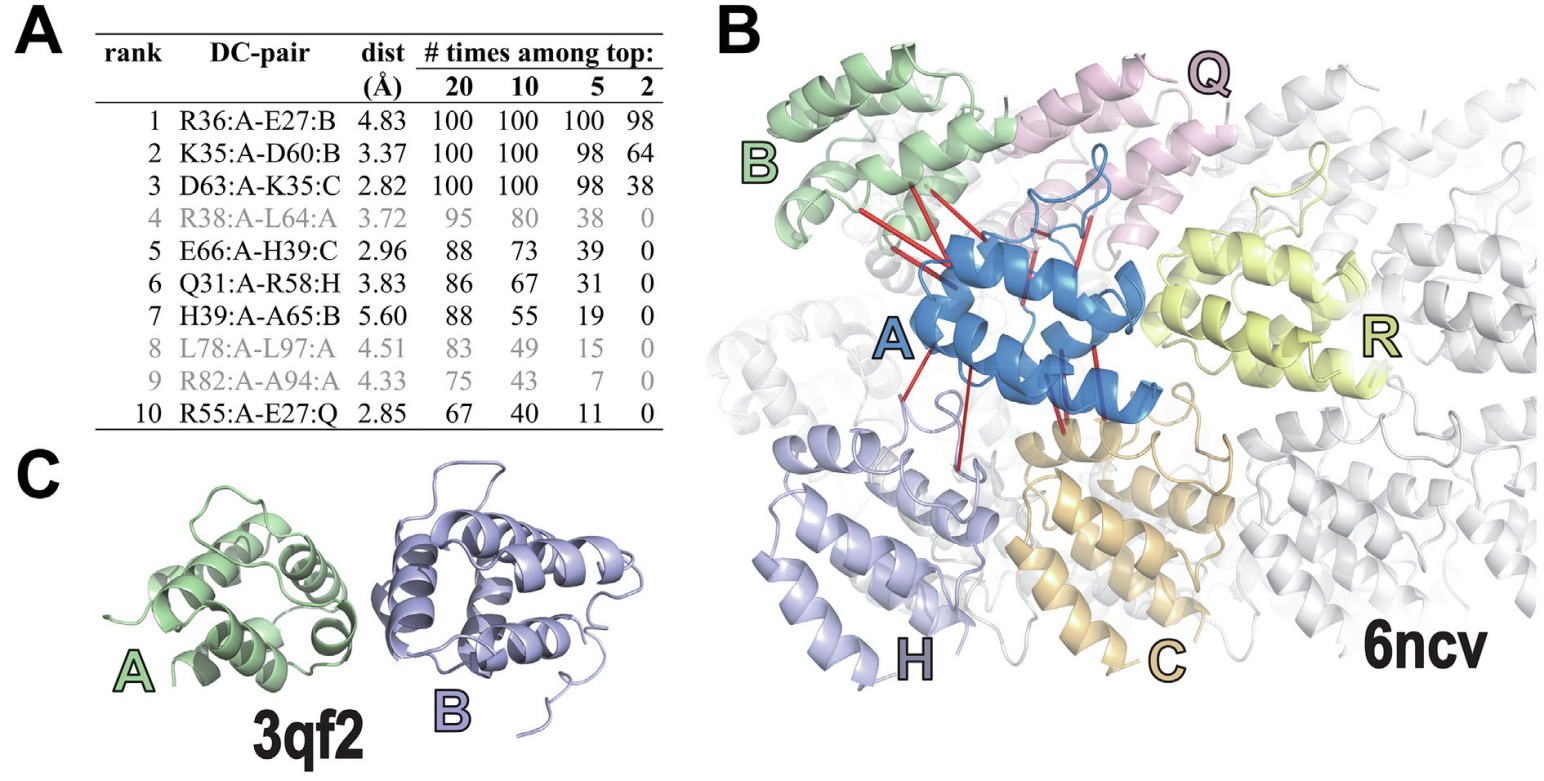
SPARC *rank* analysis of pyrin-relate death domain (DD) proteins corresponding to Table 1. A. Table of the 10 top residue pairs for the cryo-EM structure of the NLRP6 PYD filament (pdb_id: 6ncv [21]) based on sub-sampling of aligned pyrin-related sequences. SPARC robustly ranked residue pairs based on the number of times they were among the top DC-scoring (i.e., having the top average product corrected Frobenius norms) for 100 sub-samplings with replacement of the input MSA. Each sampled sub-MSA consisted of 500 sequences randomly drawn from among the 3,572 sequences in the input MSA. Seven of the 10 highest ranked pairs (those shown in black font) correspond to interactions that include contacts between adjacent death domains—suggesting that these contacts are functionally important. B. Image of the NLRP6 PYD filament cryo-EM structure. The 12 pairs that interact in trans, among the 30 highest ranked pairs, are shown as red rods. Subunits adjacent to the A subunit are colored, whereas other subunits are shown in light gray. C. Image of the NALP3 PYD crystal structure (pdb_id: 3qf2 [64]). For this structure, SPARC computes a negative value for Δ*S* suggesting that this interaction lacks biological relevance and thus may be a crystallographic artifact.

Table 1 shows the results from a *rank* analysis of pyrin-related death domains (PYD) [16], [17]; for clarity, results are given for only 6 out of 18 structures represented in the input MSA. Cryo-EM structures of the human NLRP6 PYD (pdb_id: 6ncv [21]) and of the mouse ASC PYD (pdb_id: 2n1f [22]) obtained the highest *S*-scores of *S* = 29.1 and *S* = 23.4, respectively, for chain A. These also exhibited significant Δ*S* scores with two or more adjacent subunits within their homomeric filament complexes, suggesting that these 3D contacts are biologically relevant. Indeed, Δ*S* scores of 7.6 to 12.9 for contacts with four adjacent NLRP6 PYD domains (Fig. 1) strongly support the biological relevance of this complex. In contrast, several homodimeric X-ray crystal structures (4ewi, 5h7n and 3qf2) had both lower *S*-scores (<20) and negative or barely positive Δ*S* scores (-0.9 to 0.2), suggesting that their homomeric interfaces lack biological relevance, and instead may merely be crystallographic artifacts or duplicate copies of a monomer within the unit cell. Both the NLRP6 and ASC PYD filaments exhibited a negative Δ*S* score with one other adjacent subunit, suggesting that this contact may fail to play a significant functional role.

**Table 1:** SPARC ranking of pyrin-related death domain structures by STARC *S*-score. Eighteen proteins of known structure were identified among 3,572 pyrin domain aligned sequences, 6 of which are shown. Search parameters: *r* = 4.0 Å; *m* = 5. See Table 2 for parameter definitions. A colon between two chain designations (e.g., A:C) indicates that *S* was computed using, for each residue pair, the shorter of the internal versus the homodimeric 3D distances (e.g., the A-to-A versus the A-to-C residue distances).

**pdbid**	**chain(s)**	***S***	***L***	***D***	***X***	***d***	***F***	**Δ*S***	**resolution**	**method**	**Description**
6ncv	A:C	42.0	1977	111	141	60	1.9	12.9	3.7 Å	cryo-EM	NLRP6 filament
6ncv	A:B	41.5	1977	111	141	60	1.9	12.4	3.7 Å	cryo-EM	NLRP6 filament
6ncv	A:H	37.1	1977	107	141	55	1.9	8.0	3.7 Å	cryo-EM	NLRP6 filament
6ncv	A:Q	36.6	1977	107	154	57	2.0	7.6	3.7 Å	cryo-EM	NLRP6 filament
2n1f	A:B	35.2	1977	98	141	53	1.8	11.8	4.0 Å	cryo-EM	ASC filament
6ncv	A	29.1	1977	92	141	46	1.9		3.7 Å	cryo-EM	NLRP6 filament
2n1f	A:G	28.2	1977	87	181	50	2.3	4.8	4.0 Å	cryo-EM	ASC filament
6ncv	A:R	27.7	1977	102	141	47	1.9	−1.4	3.7 Å	cryo-EM	NLRP6 filament
:	:	:	:	:	:	:	:	:	:	:	:
2n1f	A	23.4	1977	80	181	44	2.3		4.0 Å	cryo-EM	ASC filament
:	:	:	:	:	:	:	:	:			
2n1f	A:H	22.7	1977	88	141	41	1.8	−0.7	4.0 Å	cryo-EM	ASC filament
:	:	:	:	:	:	:	:	:	:	:	:
4ewi	A	19.4	2045	103	151	42	1.9		2.28 Å	X-ray	NLRP4
3qf2	A	18.9	2045	101	187	45	2.4		1.7 Å	X-ray	NALP3
4ewi	A:B	18.7	2045	107	179	45	2.3	−0.6	2.28 Å	X-ray	NLRP4
5h7n	A:B	18.2	2042	100	185	44	2.4	0.2	1.85 Å	X-ray	NLRP12
5h7n	A	18.0	2042	97	185	43	2.4		1.85 Å	X-ray	NLRP12
3qf2	A:B	18.0	2045	105	187	45	2.4	−0.9	1.7 Å	X-ray	NALP3
:	:	:	:	:	:	:	:	:			
2m5v	A	9.0	1977	91	183	31	2.3		n.a.	NMR	NLRP10

**Table 2:** List of variables defined for STARC *S*-scores.

Symbol	Definition
*L*	Total number of MSA column pairs used
*r*	Maximum 3D distance used to define contacting residue pairs (default: 4 Å)
*D*	Number of contacting pairs, i.e. distinguished elements
*X*	Optimum cut point (as defined by STARC) for partitioning an array of length *L*
*d*	Number of left-distinguished elements, i.e. contacting pairs to the left of the cut point *X* (inclusive)
*m*	Minimum sequence separation between residue pairs in query protein of known structure
ℓ	The length of the input MSA
*F*	F=X÷l indicates how spread-out is the value of *X* relative to the MSA length
*S*	-log_10_*P*, where *P* corresponds to the estimated probability after correcting for multiple tests
Δ*S*	Change in the value of *S* upon the inclusion of interactions between homomeric interfaces

Because SPARC focuses on the highest DC-scoring pairs, it is important to confirm both that each pair’s ranking (based on its DC-score) is sufficiently reproducible and that the corresponding residues observed within each protein of interest contribute positively to such scores. To address the first concern, SPARC performs DCA multiple times using subsampled sets of aligned sequences to construct a consensus ranking of the column pairs. Obtaining a consistently high DC-score for a pair of columns in an MSA implies that certain residue pairs at those positions occur more often than expected by chance and others less often; these are termed ‘elevated’ and ‘reduced’ pairs, respectively. Presumably, reduced residue pairs are being negatively selected against; if so, then proteins harboring such pairs presumably lack the structural/functional constraints detected by SPARC at those positions. Hence, SPARC addresses the second concern by differentiating between elevated and reduced residue pairs using Fisher’s exact test [23], [24] for positive and negative correlation between each specific residue pair at high DC-scoring column positions. Thus, not all residue pairs at high scoring positions in all proteins are positively correlated. For example, Arg and Glu are observed significantly less often than expected by chance (one-tail *p* = 0.001) for the top DC-scoring pair in pyrin-related death domains; hence, the corresponding R36:A-E27:B pair for NLRP6 (Fig. 1A) presumably is selected against. In stark contrast, the residues observed for the 2nd and 3rd top DC-scoring pairs (K35:A-D60:B and K35:A-D63:B) occur significantly more often than expected (*p* = 1.9x10^-28^ and *p* = 7.1x10^-29^, respectively). Prior to performing this test, residue counts are down weighted for sequence redundancy and the adjusted counts are rounded to the nearest integer.

### 2.3 Identifying direct couplings across a heteromeric interface

In the *hetmer* mode, SPARC computes the statistical significance of the correspondence between direct couplings and 3D contacts across adjacent heteromeric subunits. For this, each aligned sequence needs to be labeled with its NCBI taxonomy ID (tax_id), which can be done using our AddPhylum program (see Methods). SPARC uses tax_ids to ensure that each heteromeric subunit pair is from the same species. Unlike direct couplings across homomeric interfaces, which requires a single MSA as described above, analyses across heteromeric interfaces requires two MSAs, one for each subunit. To obtain an MSA of likely orthologs among an aligned set of homologs for each subunit and each species, SPARC selects the sequence with the highest pairwise score against the corresponding sequence from the structural coordinate file provided as the query. Some heteromeric complexes may be absent from many species, which therefore lack one or both orthologs. To help identify such cases, SPARC outputs, for each subunit, a histogram of the pairwise scores between the structural sequence and the candidate orthologs—with scores for true orthologs tending to follow a unimodal distribution that is approximately normal.

**Fig. 2:**
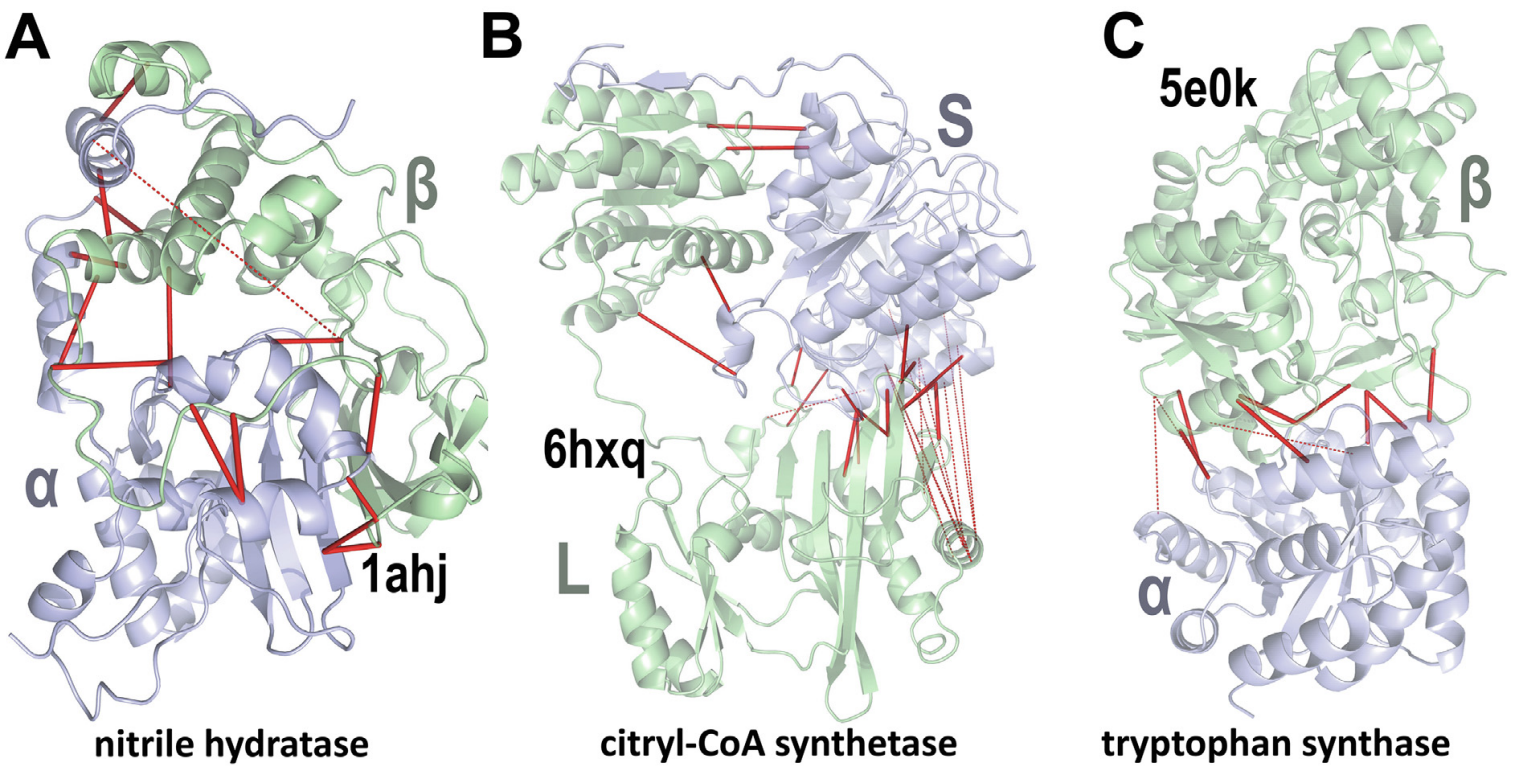
The highest DC-scoring residue pairs across heteromeric subunits for three enzyme complexes. Solid and dashed red lines correspond to DC-pairs separated by ≤ 10 Å and > 10 Å, respectively. A. The 15 highest DC-scoring residue pairs between the nitrile hydratase (pdb_id: 1ahj [65]) α and β subunits. SPARC assigns an *S*-score of ${-\text{log}}_{10}\left(p\right)=21.1\text{dits}$ (48.6 nats) to the correspondence between DC-scores and 3D heteromeric interactions within *r* ≤ 4.0 Å. B. The 27 highest DC-scoring pairs between the citryl-CoA synthetase (pdb_id: 6hxq [66]) large (L) and small (S) subunits. *S* = 42.9 dits (98.8 nats). The cluster of > 10 Å DC-pairs (dashed lines on the right) suggests that these regions may undergo conformational changes that bring them into contact. C. The 11 highest DC-scoring pairs between the tryptophan synthase (pdb_id: 5e0k [67]) α and β subunits. *S* = 21.1 dits (48.6 nats).

Table 3 shows the results for 12 heteromeric protein complexes. Anecdotally, we find that some enzymes forming a functional heterodimeric complex exhibit highly significant direct couplings at the heteromeric interface (shown in bold text in the table), whereas transient regulatory interactions tend to be marginally significant at best. Of course, computed *S*-scores also depend on other factors (see Discussion), such as the number of aligned sequences included in the analysis (as larger alignments provide more accurate DC-scores and thus a stronger signal), and the extent to which the heteromeric complex is conserved across diverse organisms. Hence, a negative result does not exclude the possibility of subtle co-evolving residues at the heteromeric interface. SPARC generates PyMOL scripts showing the structural locations of the highest DC-scoring pairs, as illustrated for three enzyme complexes in Fig. 2. The high *S*-scores observed for these enzymes strongly support the existence of co-evolving 3D contacts among *trans*-interacting residues.

**Table 3:** SPARC analysis of heteromeric interactions. Search parameters: *r* = 5.0; *m* = 5.

**name**	**pdb_id**	**Å**	**chains**	***S***	***L***	***D***	***X***	***d***	***F***	**# seqs**	ℓ_1_	ℓ_2_
AMPA-type glu receptor	5fwy	2.12	A:B	0.3	86,925	70	78,250	67	114.2	450	288	307
cdc42 GAP	1grn	2.1	A:B	3.5	30,590	72	1,547	16	4.0	2,175	161	190
cyclin E1-CDK2	1w98	2.15	A:B	0.0	48,039	70	11,478	17	21.9	2,486	240	201
**citryl-coa_synthetase**	**6hxq**	**2.91**	**A:B**	**42.9**	**83,280**	**97**	**727**	**35**	**1.1**	**17,361**	**240**	**349**
guanylate cyclase	3uvy	2.02	A:B	5.2	26,250	69	6,097	36	16.3	1,810	151	175
hemoglobin A2	1si4	2.2	A:D	4.0	18,496	23	6,912	19	31.9	984	135	137
**hydroquinone dioxygenase**	**5m4o**	**2.1**	**C:D**	**11.3**	**49,298**	**208**	**2626**	**44**	**5.3**	**340**	**157**	**321**
**nitrile hydratase**	**1ahj**	**2.65**	**A:B**	**21.1**	**37,943**	**249**	**6,592**	**110**	**16.5**	**2,057**	**186**	**204**
Rab1 GAP	4hlq	3.3	A:B	3.0	41,984	84	4,226	24	60.8	2,527	224	159
Ras RasGAP	1wq1	2.5	R:G	5.0	41,310	101	9,239	45	21.8	2,105	164	256
**SoxAX cytochrome**	**1 h32**	**1.5**	**A:B**	**18.6**	**15,836**	**51**	**168**	**17**	**0.5**	**1,094**	**148**	**110**
**tryptophan synthase**	**5e0k**	**2.76**	**A:B**	**21.1**	**70,022**	**49**	**342**	**16**	**0.5**	**21,450**	**229**	**315**

### 2.4 SPARC analyses of molecular dynamics simulated structures

The *simul* mode is one of eight modes for characterizing structural features over a time series of MD simulated structures. It computes *S*-scores at a series of time points during a simulation. This provides a sense of whether the evolving structure is departing from or converging toward conformations for which more directly coupled residue pairs make contact (i.e., toward conformations likely to be more relevant biologically and thus worthwhile examining more closely). In the *simul* mode, SPARC outputs *S*-scores computed as described for the *rank* mode above. This allows comparisons among both simulated and empirically based conformations and among various ligand-bound states (e.g., ATP- vs ADP-bound vs unbound states).

The remaining SPARC modes characterize specific types of residue interactions during an MD simulation. For these, output files are generated to visualize changes in structural interactions. Note, however, SPARC’s focus is not on simulating the dynamic motion of a protein complex per se, for which many existing programs are already available. Instead, SPARC aims to identify interactions involving pattern residues and high DC-scoring residue pairs that are congruent with current biochemical knowledge; this can suggest molecular mechanisms that explain why certain residues are subject to strong constraints.

### 2.5 Characterizing functional residue interactions in simulated structures

SPARC’s *sc2sc*, *sc2bb, sc2sb*, *dist*, *correl*, *bb2bb,* and *sipris*, modes primarily characterize residue 3D interactions for MD simulated structures over time (though they can also take as input an empirically based or AI predicted structures). For all these modes, SPARC, when performed in conjunction with a DARC analysis, reveals the category to which each interacting residue belongs and visualizes their structural locations. As illustrated in the following subsections, such analyses can provide mechanistic clues into the roles of BPPS-defined residues and of top DC-scoring residue pairs that are distinctive of a given protein functional subgroup.

#### 2.5.1 sidechain-to-residue and sidechain-to-heteroatom interactions

In the *sc2sc, sc2bb,* and *sc2sb* modes and in the *dist* mode, SPARC searches for sidechain-to-residue and (primarily) residue-to-heteroatom hydrogen bond interactions, respectively. We illustrate these analyses using the *bacterial enhancer binding protein (bEBP)* [25], [26] *NtrC1* from the extreme hyperthermophile *Aquifex aeolicus* [27]. NtrC1 activates transcription by remodeling RNA polymerase (RNAP) containing the sigma factor σ^54^ [26], [28]. When saturated with Mg-ATP or Mg-ADP, NtrC1 forms a symmetric, homoheptameric complex [29], but at slightly sub-stoichiometric amounts of the ground or transition state ATP analogs Mg-ADP-BeF_3_ or Mg-ADP-AlF_3_ it forms an asymmetric, homohexameric gapped ring [29]. The hexameric form is believed to be the functional form because it is seen when the transition state analog traps a bEBP ATPase in complex with σ^54^ [29], RNAP- σ^54^ [30], or RNAP- σ^54^-promoter [31].

**Fig. 3:**
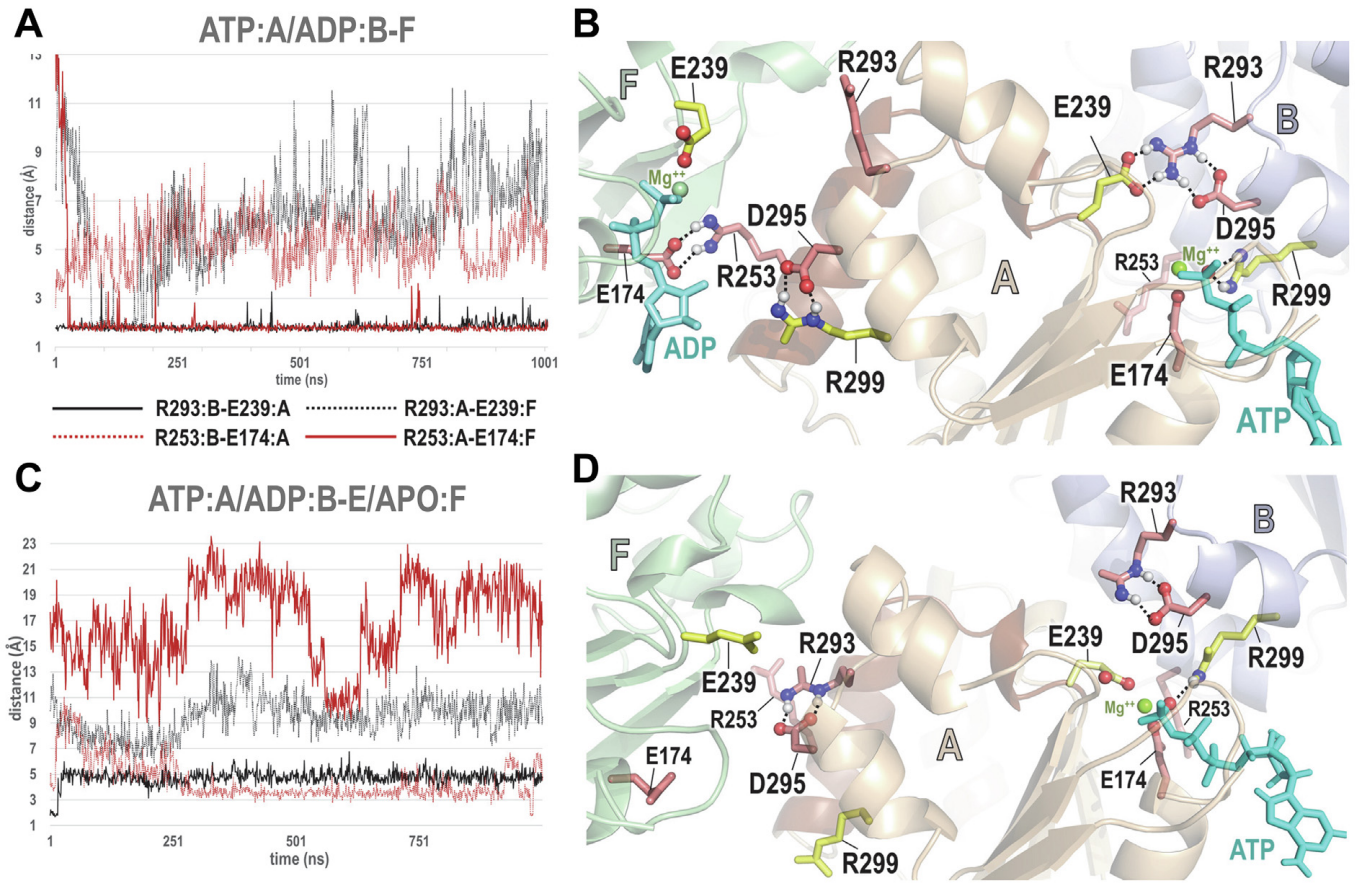
SPARC *sc2sc* analysis of MD simulated NtrC1 hexameric structures. Simulations were performed over 1 μs and were based on pdb coordinate file 4ly6 [33]. Sidechains of AAA + and bEBP associated residues are colored yellow and salmon, respectively. The plots and figures correspond to interactions at the F:A and A:B interfaces within the hexameric complex. In all cases, ADP is bound to subunits B-E. A. SPARC plot, as a function of time, of the trans interaction distances between R293:B and E239:A and between R253:A and E174:F when ATP is bound to subunit A and ADP to subunit F. B. An NtrC1 simulated structure corresponding to panel A. ATP hydrolysis may be prevented when ADP is bound to the F subunit due to the trans salt bridge between R293:B and the catalytic base, E239:A, in conjunction with a D295:B-R293:B salt bridge. The salt bridge between R299:A (the AAA + R-finger) and D295:A prevents R299:A from coordinating with ADP, whereas the R253:A-E174:F salt bridge prevents E174:F from coordinating with Mg^++^. Together these interactions may facilitate expulsion of ADP from the F subunit. Red and yellow sidechains correspond to residues distinctive of the bEBP family and the AAA + superfamily, respectively. C. Time series plot for the same residue pairs as in panel A but with ATP bound to subunit A and with subunit F in the apo state. D. NtrC1 simulated structure corresponding to panel C. Disruption of the R253:A-E174:F salt bridge may facilitate conformational changes at the A:B interface via the (dark brown) helix connected to the Walker B region, which harbors E239:A, the catalytic base. These changes reposition E239 to presumably facilitate ATP hydrolysis.

SPARC analyses suggest that NtrC1 may hydrolyze ATP only upon ADP release from an adjacent subunit. SPARC *sc2sc* and *dist* analyses of simulated NtrC1 hexameric structures over a 1 μs time course suggest a possible mechanism involving alternative interactions among residues that are distinctive of AAA + ATPases and of bEBP-related proteins (as defined by BPPS within DARC; Fig. S1). When ATP is bound to, say, the ‘A’ subunit, and ADP to other subunits within the NtrC1 hexameric complex (denoted as ATP:A/ADP:B-F), the bEBP-residue R293:B forms a salt bridge both with D295:B and, notably, with the AAA + catalytic base, E239:A, pulling it away from the γ-phosphate of ATP and thereby presumably inhibiting ATP hydrolysis (Fig. 3A,B). This interaction appears to require repositioning of a helix in subunit A whose N-terminal end is attached to the Walker B region harboring E239:A. This repositioning may be facilitated by formation (across subunit A’s interface with subunit F) of a salt bridge between two other bEBP-residues: R253:A, located within the helix, and E174:F, which directly follows the Walker A catalytic lysine residue, K173:F. In this state, the γ-phosphate of ATP bound to subunit A interacts with the AAA + *trans*-acting R-finger R299:B, whereas at the F:A interface, R299:A forms a salt bridge with the bEBP-residue D295:A—thereby sequestering R299:A away from ADP, which may then be more easily expelled from the F subunit. A SPARC *dist* analysis reveals that formation of the E174:F-R253:A salt bridge also repositions E174:F away from the ADP-associated Mg^++^ ion, which likewise may facilitate ADP release—considering that, in GTPases, release of Mg^++^ induces a 460-fold increase in the nucleotide dissociation rate [32]. Conversely, E174 may play a role in ATP hydrolysis analogous to the role of the Walker B aspartate residue by coordinating with and thereby stabilizing the nucleotide-bound Mg^++^ ion. Once ADP is removed from subunit F’s catalytic site, these salt bridge interactions are disrupted (Fig. 3C,D), thereby repositioning for hydrolysis subunit A’s catalytic base E239:A. Together, these observations suggest a mechanism to prevent catalysis at the A:B interface until ADP is expelled from the F subunit. These interactions, which were not observed in crystal structures, were stably maintained during 1 μs MD simulations based on the 4ly6 hexameric structure [33] (Fig. 3A,C). MD simulations of other nucleotide bound states (e.g., 3ATP/3ADP, 3ATP/2ADP/APO, etc.) failed to suggest such a clear-cut hypothesis regarding underlying mechanisms. The reason for this is currently unknown, though similar analyses of the complete NtrC1/RNAP-σ^54^/promoter DNA complex should provide more insight.

#### 2.5.2 Correlated formation and disruption of interacting residue pairs

In the *correl* mode, SPARC’s searches for correlated formation and disruption of hydrogen bonds between residues by performing a two tailed Fisher’s exact test on a contingency table to identify deviations from what would be expected by chance given the marginal numbers of interactions and separations for each residue pair. Note, however, that the estimated *p*-values are an invalid measure of statistical significance, because we cannot assume that the time series data points are sampled independently. Instead, SPARC uses *p*-values merely to rank each pair of residue interactions.

**Fig. 4:**
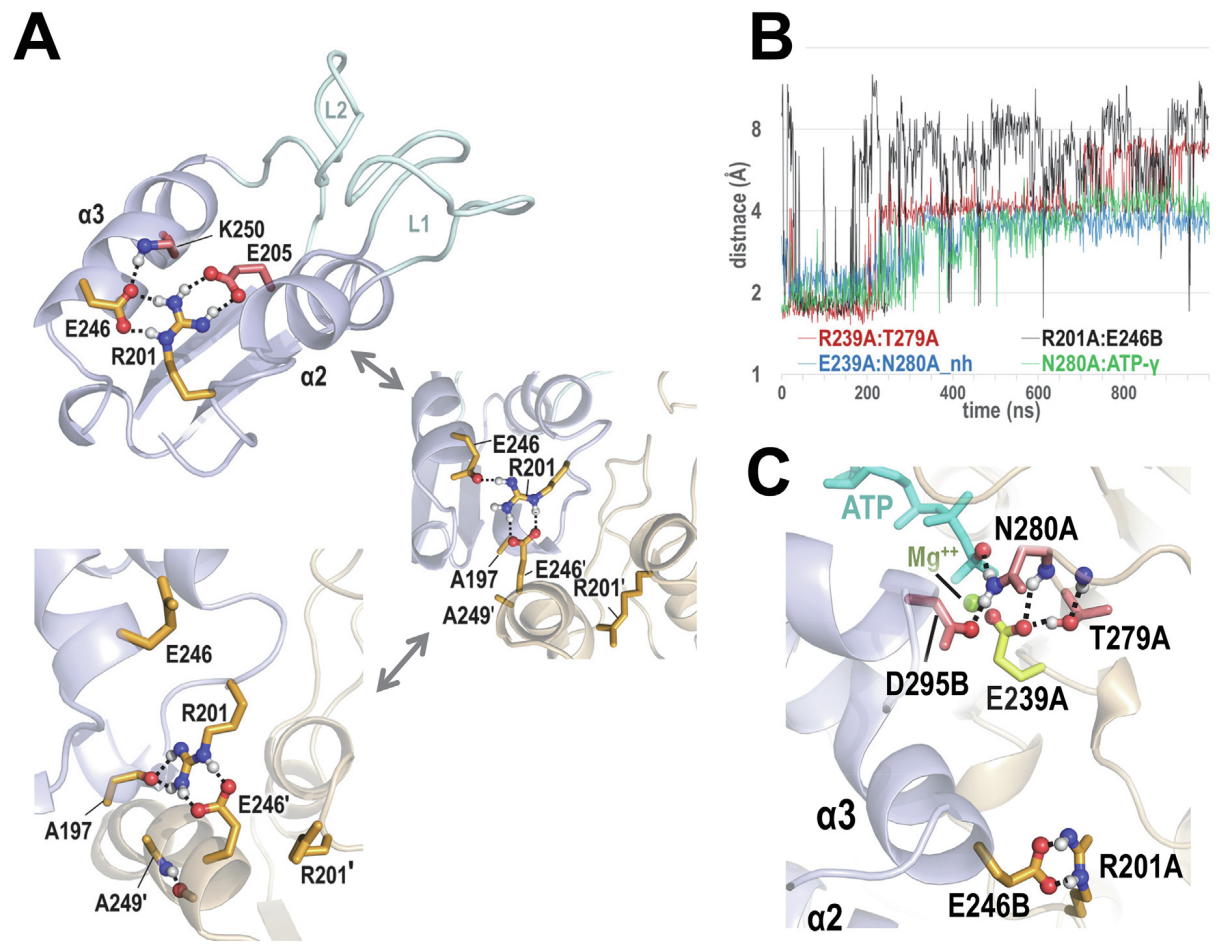
Potential allosteric coupling of catalytic residues with the R201:A-E246:B residue pair associated with the α2 and α3 helices, which are linked to the σ^54^-binding L1 and L2 loops. A. Representative *cis*-to-trans conformational switch associated with two bEBP-residues, K250 and E205 (salmon-colored sidechains), and with two high DC-scoring pairs, E246-R201 and A197-A249 (orange sidechains) (see Table 4). R201 and E246 switches between interacting in cis and trans (as shown). B. During a 1 µs MD simulation of the NtrC1 hexameric complex in the 1ATP/4ADP/APO state, the formation and dissociation of the R201:A-E246:B salt bridge (black line) is correlated with the formation and subsequent dissociation of a cluster of interacting residues at the ATP-bound active site (colored lines). The 84 ns time point corresponds to the structure shown in panel C. C. Structural locations of correlated interacting residues for the 84 ns time point in panel B.

Assuming that the NtrC1 hexameric complex in the 1ATP + 4ADP + APO state is poised to couple ATP hydrolysis to remodeling of RNAP-σ^54^, we looked for such correlations during a 1 µs MD simulation. This identified two residue pairs often undergoing correlated formation and disruption of hydrogen bonds with other pairs: an R201-E246 *cis*-to-trans switch and the disruption and formation of a E239-T279 hydrogen bond, where R201-E246 corresponds to one of the highest DC-scoring pairs (Table 4), E239 corresponds to the AAA + catalytic base and T279 to the AAA + sensor 1 motif. A closer investigation using the *sc2sc*, *sc2bb*, and *dist* modes revealed that, for the ATP-bound A subunit, the R201-E246 *cis*-to-trans switch (Fig. 4A,B) correlates with the disruption and formation of a E239-T279 hydrogen bond (Fig. 4B,C) and that formation of the R201-E246-trans hydrogen bond is correlated with the interaction of another sensor 1 residue, N280, with both E239 and the γ-phosphate group of ATP (Fig. 4B). Hence, we hypothesize that formation of the R201-E246-trans salt bridge, in conjunction with formation of interactions among E239, T279, N280, D295, and ATP, may prime the active site for hydrolysis (Fig. 4C). This and the location of R201 and E246 within the α2 and α3 helices, which are associated with the L1 and L2 σ^54^-binding loops, suggests an allosteric mechanism for coupling ATP hydrolysis to remodeling of the RNAP-σ^54^ complex.

**Table 4:** Top 15 highest DC-scoring residue pairs identified by SPARC for NtrC1 based on a bEBP family MSA.

rank	residue pair^a^		description or comment	shown in	% sampled among top^b^			
	site 1	site 2		figures	20	10	5	2
1	A197	A249	May avoid steric clashes	yes	100	100	93	71
2	R201	E246	Electrostatic contact noted in [27]	yes	100	100	86	38
*3*	*K327*	*K360*	*K-to-K is significantly reduced*	*–*	100	100	88	26
*4*	*F236*	*A278*	*Not distinctive of bEBP family*	*–*	*100*	*100*	*87*	*26*
5	I153	V176	Contact between helices 1 and 0	–	100	100	79	33
6	F227	V254	Contact between helix 3 and L1 helix	–	100	96	30	4.3
7	E256	K360	E256 binds to Sensor-2 Arginine	–	100	83	20	0.7
8	K155	E371	Contact between adjacent domains	–	100	78	10	0.6
9	***W352***	*V362*	V362 packs against W352 and E358	–	100	71	6.2	0.3
10	E242	R281	Bridge next to E242-R293 bridge	–	100	51	0.4	0
11	**E205**	**K250**	Electrostatic contact noted in [27]	yes	99	33	0.1	0
*12*	*F339*	*I363*	*Not significantly elevated*		*94*	*30*	*0.2*	*0*
*13*	*I318*	*Q344*	*Not significantly elevated*	*–*	*98*	*19*	*0.1*	*0*
14	**E174**	**R253**	trans salt bridge near Walker B D238	yes	88	10	0	0
15	*V171*	*I307*	Contact between strand 5 and P-loop	–	84	12	0.3	0

a Residues in bold were also identified by BPPS as among the most distinctive of bEBPs. Rows in italicized, light gray font correspond to pairs that are not significantly elevated and thus not among those residue pairs subject to constraints or that are also among the highest scoring pairs in other AAA + proteins and thus are not distinctive of bEBPs.

b Subsampling: each of 1,000 subsamples of 2,500 aligned sequences randomly drawn from the bEBP MSA (95,469 sequences) were used to compute DC-scores. The last 4 columns give the percentage of samplings for which the residue pair in each row was among the top 20,10, 5 or 2 highest DC-scoring out of 17,085 pairs.

#### 2.5.3 backbone-to-backbone interactions

**Fig. 5:**
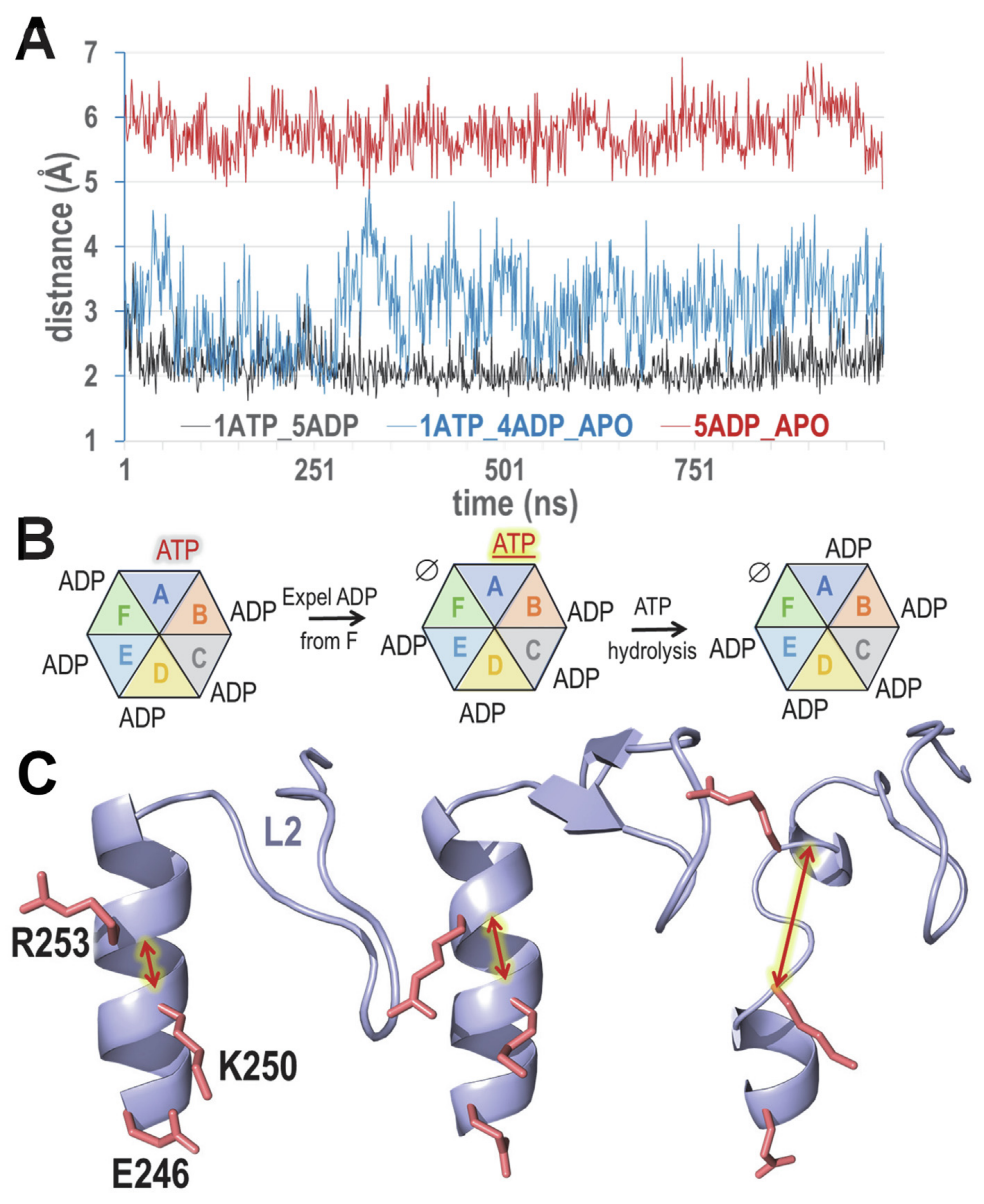
Conformational changes associated with the α3 helix of the NtrC1 A subunit during a 1 μs MD simulation. A. SPARC analyses of backbone-to-backbone (*bb2bb*) interaction distances between residue K250 and the residue adjoined to R253, namely V254. Distances are plotted as a function of time for the three nucleotide bound states of the NtrC1 homohexamer shown in panel B. Note how the black and red plots are quite distinct. B. Schematic representation of the three hexameric nucleotide bound states. C. Representative conformations of the α3 helix and L2 loop of the A subunit for the hexameric states shown directly above each image in panel B. When ATP is bound to the A subunit and ADP to the other five subunits, the α3 helix of subunit A is well formed. However, when ADP is removed from the F subunit, a kink tends to form in subunits A’s α3 helix. The helix α3 kink becomes more extensive upon hydrolysis of ATP to ADP. Because the α3 helix is attached to the N-terminal end of the L2 loop, the kink may facilitate restructuring of σ^54^ bound to the L2 loop. Salmon colored sidechains correspond to BPPS-defined residues distinctive of bEBPs (see Fig. S1).

If ATP hydrolysis occurs when in the 1ATP/4ADP/APO state, then this would transition the complex into the 5ADP/APO state, for which we also performed a 1 μs MD simulation. SPARC *bb2bb* time analyses of all three states (Fig. 5A) reveal striking conformational differences in the α3 helix, which directly precedes the σ^54^-binding L2 loop: Upon release of ADP from subunit F, this helix begins to develop a kink in helix α3 that becomes more extreme upon hydrolysis of ATP (Fig. 5B,C). The α3 helix harbors R253, which forms with E174 a salt bridge that often undergoes correlated formation and disruption of hydrogen bonds with other pairs, including R201:E246. The α3 helix also harbors two other residues subject to strong constraints: K250, which is distinctive of bEBP-related proteins, and E246, which, as mentioned above, forms one of the highest DC-scoring pairs with R201 (Table 4). Moreover, as indicated in Fig. 4, the salt bridge formed by the E246-R201 DC-pair often switches between cis and trans interactions, where, for the trans interaction, these residues can form hydrogen bonds with the highest DC-scoring and *trans*-interacting pair A197-A249 (Table 4; Fig. 4A, bottom left image). The short alanine sidechains on these residues may facilitate the E246-R201 *cis*-to-trans switch by avoiding the steric clashes that might occur with longer sidechains. R201 is near the N-terminal end of the α2 helix, which harbors both another bEBP-specific residue, E205, with which R201 also interacts (Fig. 4A), and the L1 loop that also binds to σ^54^. Together, these structural features suggest an allosteric mechanism for coupling ATP-hydrolysis to remodeling of RNAP-σ^54^.

#### 2.5.4 Hydrogen bond networks involving pattern residues

We used SPARC, in conjunction with DARC, to find hydrogen bond networks among BPPS-defined pattern residues for the *E. coli* helicase DnaBC complex, which opens the replication fork during DNA replication. To identify pattern residues, we ran DARC on an MSA of 675,713 sequences belonging to the RecA-like superfamily, using as the query the *E. coli* DnaB protein. This identified pattern residues most distinctive both of the RecA superfamily and of the DnaB-like family, as highlighted in Fig. S2. To further characterize these residues, we performed MD simulations based on a recent cryo-EM structure of the *E. coli* DnaBC complex [34]. In this complex, the DnaB helicase and the DnaC AAA + ATPase each consist of six subunits forming a ring-shaped homohexamer with each ring packed against the other and with a central pore, through which single stranded DNA (ssDNA) is thread.

**Fig. 6:**
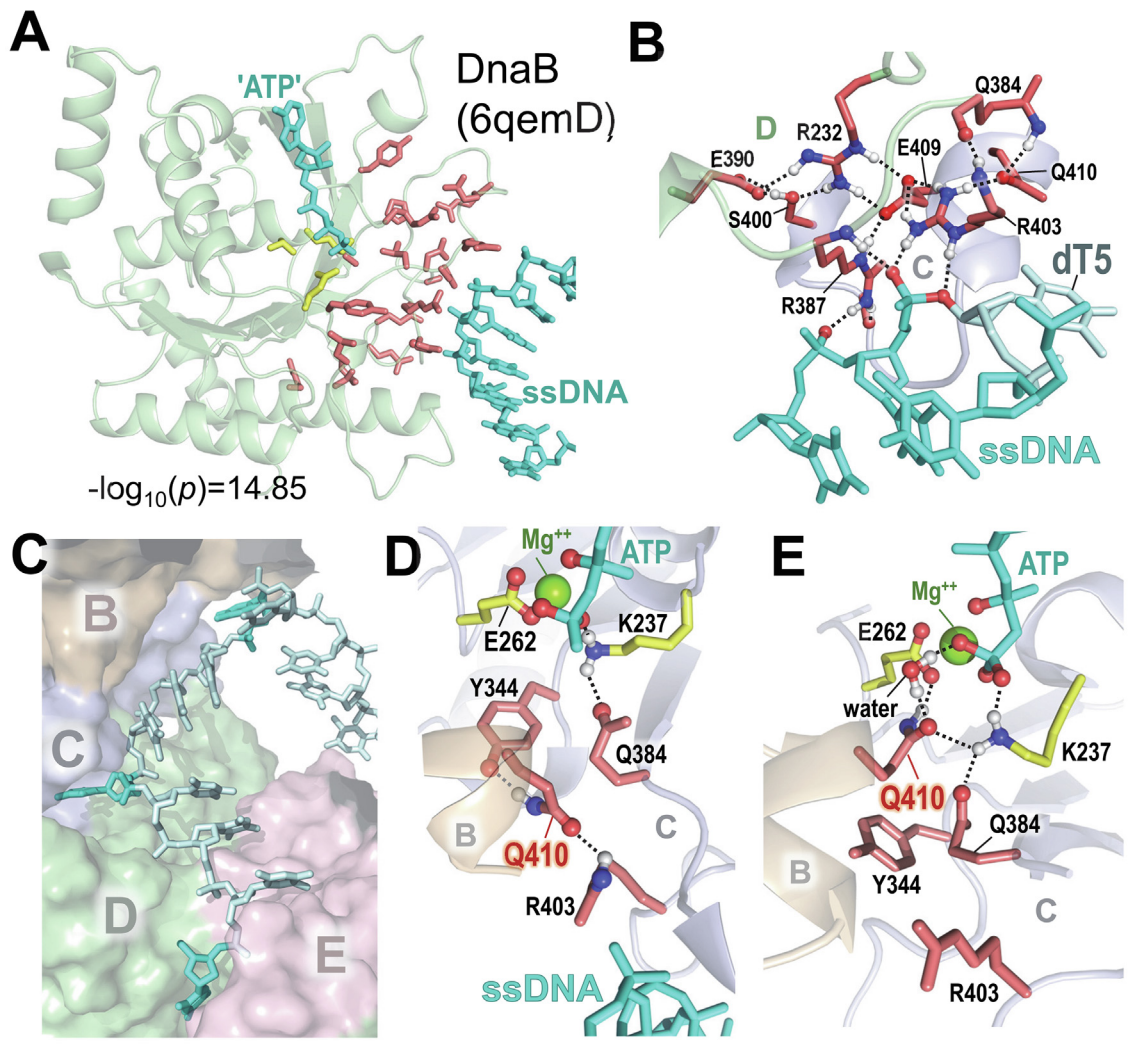
Residues distinctive of DnaB-like and RecA-like proteins within an MD simulated structure of the DnaBC complex bound to ATP and ssDNA. A. BPPS-defined residues (see Fig. S2) distinctive of DnaB-like proteins tend to cluster between ssDNA and ATP within the cryo-EM structure of the DnaBC complex (pdb_id: 6qem [34]). Shown are highly significantly clustered ($p=1.4\times 10^{-15}$) DnaB-like residues (red sidechains) that were identified by SPARC (using the *sipris* mode) along with three catalytic residues (K237, T238, and E262; yellow sidechains) distinctive of the RecA-like superfamily. B. A network of hydrogen bonds formed among eight DnaB-like residues at the C:D interface during a 1 μs MD simulation. This network includes hydrogen bonds to ssDNA and is associated with a flipped-out thymidine base (dT5; palecyan). Formation of this network may facilitate the base flip. A similar flipped-out base occurs at the D:E interface (as shown in panel C). C. Two thymidine bases within the ssDNA that, during the simulation, flip out and insert into cavities associated with the C:D and D:E interfaces within the homohexameric complex and a third flipped out thymidine base that is near but not within the B:C cavity. Flipped out bases are colored cyan; other bases are colored palecyan. D. MD simulated (23 ns time point) conformation of residue Q410 at the B:C interface while interacting with ssDNA-associated R403. E. Later during the simulation (683 ns time point) Q410 has flipped around to interact with the ATP binding site. Q410 interacts both with a sidechain oxygen atom of the putative catalytic base, E262, and with a buried water oxygen atom, which could attack the γ-phosphorous atom to mediate ATP hydrolysis—perhaps in conjunction with the 3rd flipped base fully exiting the B:C cavity. E262 also coordinates with the ATP-bound Mg^++^ ion. Residues distinctive of the RecA-like superfamily in panels D and E are shown with yellow sidechains.

Using SPARC’s *sipris* mode we found that, within the DnaBC complex, residues distinguishing DnaB-like helicases from other RecA-like proteins form a highly significant (***p* < 10^-14^**) cluster located in loop regions between the catalytic site and ssDNA (Fig. 6A). To investigate 3D interactions among these residues, we performed a 1 μs MD simulation of this complex. SPARC finds that, during the simulation, these residues can form an intricate hydrogen bond network (Fig. 6B) that is associated with a flipped out nearby base of ssDNA (Fig. 6C). Within the complex, three of the ssDNA bases flip in this way, two of which are inserted into a cavity formed at the C:D and D:E interfaces between subunits (Fig. 6C), perhaps thereby ‘grabbing’ onto the DNA strand. At these interfaces and early in the simulation at the B:C interface, one of these residues, Q410, is positioned toward the ssDNA. Later in the simulation, however, Q410:B (at the B:C interface) flips around to form hydrogen bonds with the putative catalytic base (E262:C) and with a water molecule (Fig. 6D). The water’s oxygen atom is positioned to attack the γ-phosphorous atom of ATP where one of the water’s hydrogen atoms could be taken up by Q410:B’s sidechain oxygen and the other by a γ-phosphate oxygen (Fig. 6D). If Q410:B is required for hydrolysis, then the Q410:C and Q410:D flipped ‘down’ states may prevent premature ATP hydrolysis at the C:D and D:E interfaces. Hence, these conformational states may reflect mechanistic features coupling ATP hydrolysis to translocation of the helicase along DNA. These interactions are evident during an MD simulation, but not in the cryo-EM structure used to set up the simulation (pdb_id: 6qem) [34]. Because the participating residues are conserved among the 86,801 DnaB-like proteins used in our analysis, which represent 38 bacterial phyla, these findings may provide a structural context for rational drug design of broad-spectrum antibiotics.

## 3 Discussion

SPARC and our overall approach focus on large superfamilies that have functionally diverged into subgroups, each of which has its own, characteristic constraints. This provides the best opportunity to glean clues to underlying molecular mechanisms by hierarchically categorizing constraints. Although the statistical power of such an analysis depends on the number of aligned input sequences, there are hundreds of sufficiently large superfamilies currently available, for which MSAs can be created from NCBI CDD hierarchies [35]. There is no sharp cutoff regarding the minimum number of input sequences required, though, roughly speaking, there should be at least say, 2,000 sequences given that there are 400 possible residue pairs for 20 amino acids. Conversely, statistical power will tend to converge as the data becomes sufficiently large. Ideally, SPARC should be applied to superfamilies with at least 20,000 distinct sequences or, better yet, with several hundred thousand or more sequences and with a lot of structural data.

SPARC differs from other MD analysis tools, as it specifically identifies and structurally visualizes 3D clusters and interactions involving constrained residues and top DC-scoring residue pairs, while also assessing each structure’s potential biological relevance; though it can also be applied to empirically based structures. SPARC also computes *S* scores for heteromeric interfaces within multimeric complexes and allows a user to investigate the most interesting interactions involving individual residues or residue pairs in greater depth. It incorporates more common ways to search for residue interactions and correlations (which our other programs do not) and therefore serves as a single tool to perform an extensive, multifaceted analysis. Traditional MD analysis tools like MDAnalysis [36], MDtraj [37] and Pycontact [38] also focus on low resolution metrics, such as RMSD, RMSF, radius of gyration, or solvent accessible surface area, whereas SPARC focuses on specific hydrogen bonds involving residues or residue networks subject to statistically significant constraints. Hence, SPARC performs analyses from a perspective that, to our knowledge, existing MD analysis tools do not.

Although deep learning methods can leverage vast amounts of information to perform well on shallow MSAs, they will obscure the residue constraints that SPARC seeks to characterize. Consider, for example, the top CASP12- and CASP13-winning contact and distance prediction method, RaptorX-Contact [39]. This method relies on CCMpred DC-scores, just as SPARC does, but also relies on (i) a position-specific scoring matrix, (ii) predicted secondary structure confidence scores, (iii) predicted solvent accessibility scores, and (iv) three other matrices for pairwise relationships generated by the alnstats routine in MetaPSICOV [40]. Consequently, by merging such information, RaptorX-Contact will obscure the DC-signals associated with pairwise correlations in unanticipated ways, so that a clear signal cannot be associated with a specific residue pair. This would undermine SPARC’s main objective. Nevertheless, SPARC can compute *S* scores using rankings based on contemporary deep learning DCA methods using a command line option that takes arbitrary DC-scores as input. This will estimate the statistical significance of the correspondence between the highest of these alternative DC-scoring pairs and the 3D contacts within structures, just as they do for SPARC’s built-in DC-scores.

We prefer empirically based structures over AI predicted structures, because SPARC works best using protein multimeric complexes bound to cofactors, substrate, other ligands, ions, and other, interacting subunits; these are currently unavailable for AlphaFold [41] structures—though RoseTTAFold [42] is able to model multiple subunits. Meaningful sidechain interactions and loops are another key element of SPARC analyses both for static structures and as a good starting point for MD simulations; however, AI predicted structures are reported as having inaccurate sidechains and loops and other limitations [43], [44]. Nevertheless, as AI predictions improve to include protein complexes in substrate and cofactor bound states, it will be straightforward to perform SPARC analyses on these as well.

SPARC, in conjunction with DARC, structurally characterizes residue constraints as likely determinants of protein function, leading to clues regarding possible underlying mechanisms. SPARC’s modes provide multiple perspectives on structural features associated with those constraints. We illustrated this for empirically based structures of homomeric death domain filaments and of various heterodimeric complexes. This revealed that both a cryo-EM structure of a death domain filament and certain crystal structures of heterodimeric enzymes have highly significant (interface) Δ*S* and *S* scores—suggesting that these interactions are biologically relevant. An avenue for further research is predicting heteromeric interaction sites when only the structures of the individual protomers are available. In this case, one could search over possible interfaces for the highest *S*-scoring interface, as the likely actual interface. Notably, SPARC’s ability to pair up heterodimeric subunits from distinct species automates a process that is now often performed manually.

SPARC also provides complementary information, such as whether specific residues are elevated or depressed at high DC-scoring MSA positions; this reveals whether certain residue pairs in a protein of interest are subject to the DC constraints imposed at those positions in other, related proteins. Although not described here, SPARC can be used in this way to assess the quality of predicted versus empirically based protein structures. For example, we found that AlphaFold [41] structures for GTPase domains received similar *S*-scores as did empirically based structures.

Some of the heterodimers (e.g., AMPA-type Glu receptor and cyclin E1-CDK2 in Table 3) failed to achieve significant *S*-scores. This could be due to a variety of reasons: For example, as organisms adapt to changing conditions, interactions of a regulatory protein with regulated proteins may evolve more rapidly, and thus obtain a lower *S*-score, than would a more stable, biochemically critical heteromeric enzyme interaction. Alternatively, as illustrated in [45], the correspondence between high DC-scoring residue pairs and 3D contacts may be obscured due to their association with transient interactions during folding or upon binding. Likewise, while performing its cellular function, a protein complex may cycle through alternative states, only some of which may bring together top DC-scoring pairs. SPARC could be applied, of course, to such alternative conformations if their structural coordinates were available.

DC-signals associated with subunit interactions may also fail to be conserved across members of an evolutionarily related protein family due to divergence in structure or function, leading to differences in subunit contacts, or in folding, or binding properties. This, in turn, may be due to environmental differences, among, for example, thermophilic vs cryophilic, halophilic vs osmophilic, or acidophilic vs alkaliphilic bacteria. A low *S*-score could also be due to crystallographic artifacts, to an incorrect structure, or to insufficient data to obtain a clear-cut DC signal. In any case, an insignificant *S* or Δ*S* score does not exclude the possibility of certain subunit interactions being biologically relevant—though SPARC cannot determine whether any constraints are obscured and, if so, why. (In such cases, PDBePISA [46] or 3D complex [47] might differentiate between real and artifactual low *S*-scoring interactions.) For these reasons, SPARC focuses solely on high *S* scoring structures that are more likely to exhibit interactions associated with the underlying molecular mechanisms that we seek to understand. Indeed, obtaining a highly significant *S* score for an incorrect conformation is highly improbable. This is a major advantage of performing a statistical analysis, as the *p*-value estimate does not depend on the amount of or the quality of the data, but rather on the probability of obtaining the observed result by chance alone. An incorrect protein structure or incorrect DC-scores should only lead to statistically insignificant results.

Because protein structure is dynamic, MD simulations add an important dimension to the analysis and interpretation of sequence and structural constraints and vice versa. SPARC examines, in this way, dynamic changes associated with BPPS-defined residues and clusters, and with high DC-scoring residue pairs. This provides otherwise unavailable clues regarding underlying mechanisms, leading to plausible hypotheses for experimental follow up. For example, our analysis of the NtrC1 hexamer suggests a possible mechanism to couple hydrolysis at an ATP bound subunit with nucleotide exchange at an adjacent, ADP-bound subunit: Two *trans*-interacting salt bridges involving three bEBP-specific residues and the E239 catalytic base (R293:B-E239:A and R253:A-E174:F) were stably maintained over a 1 µs simulation in the ATP:A/ ADP:B-F state. Also stably maintained in this state is a *cis*-interacting salt bridge between the AAA + R-finger R299:A and the bEBP-specific residue D295:A (R299:A-D295:A). The R293:B-E239:A salt bridge sequesters the catalytic base away from ATP-Mg^++^ bound to the active site; the R253:A-E174:F salt bridge sequesters E174:F away from its interaction with the ADP-bound Mg^++^ ion; and the D295:A-R299:A salt bridge sequesters the *trans*-acting R-finger away from its interaction with ADP bound to subunit F. However, upon release of ADP from the F subunit, these salt bridges are disrupted leading to the interaction of the catalytic base (E239:A) with ATP-Mg^++^. Together, this suggests a mechanism facilitating concurrent release of ADP from subunit F and ATP hydrolysis at subunit A.

This raises the question: How is ATP hydrolysis coupled to remodeling of the RNAP-σ54 complex? SPARC analyses of 1 µs simulated structures of NtrC1 in the ATP:A/ADP:B-E/APO:F state reveal a correlation between a *cis*-to-trans switch of the R201-E246 salt bridge and the interact of three bEBP residues with bound ATP and with the catalytic base. E246 is in the α3 helix, which attaches to the L2 loop, and R201 is at the N-terminal end of the α2 helix, which harbors the L1 loop; these loops are believed to play key roles in binding and remodeling of RNAP-σ^54^ during promoter melting [27], [28]. A SPARC comparison of the ATP:A/ADP:B-E/APO:F state with the ADP:A-E/APO:F state, which would result upon ATP hydrolysis and P_i_ release, reveals a dramatic restructuring of helix α3 in subunit A. Conformational changes associated with such collapse of helix α3 could cause loops L1 and L2 to deliver mechanical work to σ^54^. A less severe distortion in helix α3 involving these same residues was reported upon comparing crystal structures of NtrC1 ATPase saturated by ADP and a non-hydrolytic mutant saturated by ATP [27]. However, those studies with wildtype and mutant NtrC1 were in the context of symmetric, heptameric (probably nonfunctional) forms. Together, the SPARC analyses reported here provides new mechanistic clues regarding specific nucleotide states within the asymmetric, gapped hexameric (probably functional) form of NtrC1. In this way, SPARC expands on the existing glutamate-switch [48] and rigid-body-roll [27], [33] proposals for how the bEBP subclass of AAA + ATPases function. Arriving at such a new hypothesis illustrates how SPARC can be useful for experimental design.

SPARC/DARC analyses in conjunction with MD simulations likewise provided clues regarding mechanisms associated with DnaB helicases. Within the cryo-EM structure of the DnaBC complex, SPARC found, between the active site and ssDNA, a highly significant cluster of BPPS-defined residues distinctive of the DnaB family. SPARC analysis of DnaBC simulated conformations identifies a hydrogen bond network involving DnaB-like residues and associated with ssDNA flipped-out bases inserted into a cavity between DnaB subunits. SPARC also identifies a conformational switch involving Q410 that may be involved in activation of ATP hydrolysis. These structural features are hypothesized to be involved in coupling hydrolysis to translocation of the helicase along DNA.

Francis Bacon, in his book *Novum Organum* [49], describes, as the first step in applying the scientific method, the compilation of observational data, followed by the categorization of these observations and the generation of hypotheses. As illustrated here, by extensively characterizing protein sequence and structural constraints, SPARC facilitates the generation of hypotheses, from which may follow the accumulation of additional empirical results through further experimentation.

## 4 Methods

*Protein structural coordinates*. Structural coordinate files were obtained from the RCSB protein data bank (PDB) [50]. The PDB identifiers for the structures examined in this study were: 6ncv, 2nlf, 4ewi, 3qf2, 5h7n, 2m5v, 5hwy, 1grn, 1w98, 6hxq, 3uvy 1si4, 5m4o, 1ahj, 4hlq, 1wq1, 1 h32, 5e0k, 4ly6, and 6qem. Hydrogen atoms were added to these files using the Reduce program [20] version 3.3.

*MAPGAPS (version 2.0)*. MAPGAPS (Multiply Aligned Profiles for Gapped Alignment of Protein Sequences) [51] can both identify and accurately align up to a million or more sequences, taking as input a fasta-formated database file of protein sequences along with a hierarchical MSA, such as are available from the NCBI (ftp://ftp.ncbi.nlm.nih.gov/pub/mmdb/cdd/hiMSA)[52]. A hierarchical MSA consists of a set of sub-MSAs (one for each subgroup in a superfamily) and a template MSA that globally aligns the sub-MSAs to each other. From the hierarchical MSA, MAPGAPS creates a set of multiply aligned profiles, which then detect and align related database sequences, as follows: Each sequence that scores above a specified threshold against the root profile is first locally aligned against the most closely-related profile; then all of the sequences detected in this way are globally aligned using the template MSA. With sufficiently diverse sequence representation in the hierarchical MSA, the output MSA will be of comparable quality. Due to memory limitations, we split database sequences into smaller files containing no more than 250,000 sequences each and run MAPGAPS on each of these files separately, the resulting MSAs were then merged into a single MSA. We removed sequence fragments (i.e., those with > 25% deletions) and all but one sequence among those sharing ≥ 95% identity using the PurgeMSA program, which is included with MAPGAPS. We also used MAPGAPS to detect and multiply align related proteins of known structure within the NCBI pdbaa fasta file (available at: ftp.ncbi.nlm.nih.gov/blast/db/FASTA/pdbaa.gz); these were added to the MSA, and PurgeMSA was used to remove sequences identical to these sequences from the initial MSA. This allows SPARC, BPPS, and DCA to associate protein structural coordinates with corresponding sequences in the alignment.

*Jackhmmer MSAs*. We used the following six steps to create MSAs using Jackhmmer [53]. (1) We labeled NCBI nr, env_nr, and translated EST fasta-formatted sequences with their NCBI taxonomy identifiers using the AddPhylum progam, which is available with SPARC. (2) For heteromeric interface analyses, we used each subunit of a heterodimeric complex of known structure as a query in a PSI-BLAST [54] search to obtain a set of related database sequences. (3) For each of the two sets, we applied cd-hit [55] to reduce redundancy at a sequence identity threshold of 95%. (4) We used Jackhmmer to iteratively align sequences to each query. (5) We removed aligned columns with > 50% deletions and sequences that failed to match at least 95% of the retained columns. (6) For each species, SPARC retains a pair of sequences, one sequence from each MSA that is most similar to the query for that MSA; any remaining sequences for that species are removed. For the DnaB analysis, Jackhmmer was used in the same way to create an input MSA.

*SPARC/DARC*. SPARC/DARC input files included: (i) an MSA that includes proteins of known structure; (ii) corresponding 3D structural coordinate files; and (iii) a designated sequence in the MSA to serve as a query to seed the DARC analysis. DARC’s statistical models and algorithms were recently described in detail [15]. The MSA obtained was in cma format, which requires less memory than other formats. Fasta formatted MSAs may be converted to cma format using the auxiliary ConvertMSA program provided with SPARC. We first ran DARC to define the query protein family and the pattern residues distinguishing family members from other sequences based on the input MSA; this step creates rich text format ‘contrast’ alignment output files, as shown in Figs S1 and S2. Using the information obtained from DARC, we ran SPARC in various modes to structurally characterize and visualize (via PyMOL scripts) pattern residues and directly coupled residue pairs within protein structures. PyMOL is available at http://www.pymol.org/.

*DCA*. The source code for CCMpred version 0.3.2 (https://travis-ci.org/soedinglab/CCMpred) [19] was incorporated into the SPARC program. To determine whether different input MSAs rank DC-pairs consistently, a SPARC auxiliary routine performs subsampling. From the input MSA for NtrC1, this routine drew 1,000 samples of 2,500 sequences, from each of which DCA scores were computed. Between samplings, the 2,500 previously sampled sequences are replaced prior to sampling the subsequent set. The percentage of times that each residue pair was among those with the top 20, 10, 5 or 2 DC-scoring pairs are reported. The robustness of the bEBP NtrC1 analysis was further confirmed by subsampling in this way for two different full-size input MSAs: one consisting of 108,178 sequences and another consisting of 62,601 sequences.

*Molecular dynamics (MD) simulations*. MD simulations for NtrC1 were performed using AMBER18 [56]. The configuration of the protein complex and of bound nucleotide were based on the crystal structure 4ly6, which contains 4 hexameric complexes within the unit cell. Models with one apo unit were based on the hexamer corresponding to subunits A-F, five subunits of which are bound to ADP-BeFx; models without an apo unit were based on the chain G-L hexamer, for which all six subunits are bound to ADP-BeFx. Bound ADP was constructed by removing the beryllium (Be) and the three fluorine (F) atoms of ADP-BeFx; bound ATP was constructed by replacing each Be with phosphorous (P), and each F with oxygen (O). Counterions (∼0.01 M NaCl) were added to neutralize the system. The system was then solvated with TIP3P water molecules. The size of the initial system was 190 × 127 × 184 Å^3^ and contained ∼ 400,000 atoms. Minimization and equilibration stages were conducted by gradually reducing constraints on the protein and bound ligands. First, a 1000-step minimization was performed consisting of 400 steps of steepest decent and 600 steps of conjugated gradient minimization with protein and bound ligands positions constrained using a force constant of 250 kcal/mol/Å^2^. After minimization, the system temperature was increased to 300 K through two sequential runs, with 10 and 2 kcal/mol/Å^2^ constraints placed on the positions of the protein and bound ligands, respectively. First, the system was heated to 100 K for 20 ps in an NVT ensemble, and then it was slowly heated to 300 K for 100 ps at 1 atmosphere in an NPT ensemble, with a 2 fs time step, 10 Å nonbonded interaction cutoff, and SHAKE-constrained hydrogen bonds. A short 500 ps NPT simulation was then performed with no constraints prior to production simulations. Conventional MD simulations were conducted on the models for 1000 ns with periodic boundary conditions, a temperature of 300 K, a pressure of 1 atm, 2 fs time steps, 10 Å nonbonded interaction cutoff, and SHAKE-constrained hydrogen bonds. In all simulations, the protein was described by the FF14SB force field [57], the ADP and ATP by the parameters obtained from the Bryce AMBER Parameter Database [58], and the H_2_O molecules and counterions were described by the TIP3P model [59]. One thousand structures were obtained by sampling every ns during the simulation.

MD simulations for DnaBC were performed using OpenMM-7.4.1 [60] with input files created using CHARMM-GUI [61] with the OpenMM generator [61], [62] and using the CHARMM36 additive force field. The configuration of the protein complex and of bound nucleotide were based on the crystal structure 6qem, which contains the doubly hexameric DnaBC complex bound to ssDNA and with ADP bound to each of the six DnaC subunits and ADP⋅BeF_3_ bound to five of the six DnaB subunits (denoted as chains A-F, with F in the apo state). For simulations, the bound ADP⋅BeF_3_ was changed to ATP by replacing each beryllium (Be) atom with phosphorous (P), and each F with oxygen (O). We used the CHARMM-GUI default settings for adding counterions to neutralize the system and for solvation with TIP3P (explicit) water molecules. Minimization and equilibration stages were conducted using the CHARMM-GUI default input parameters. MD simulations were conducted for 1000 ns with periodic boundary conditions, a temperature of 303.15 K, a pressure of 1 atm, 2 fs time steps, 10 Å nonbonded interaction cutoff, and constraints on bonds involving hydrogen. One thousand structures were obtained by sampling every ns during the simulation.

*S-scores*. SPARC estimates of the statistical significance of the correspondence both between the highest DC-scoring pairs and 3D contacts (as in STARC) and between BPPS-defined pattern residues and spatially adjacent residue clusters within a structure (as in SIPRIS) by applying Initial Cluster Analysis [4] to compute *S* = -log_10_(*p*). In the former case, SPARC assesses the correspondence between top DC-scoring pairs and *internal* contacts alone for each chain (e.g., labeled as chain ‘A’) and between top DC-scoring pairs and both *internal* and adjacent subunit *interface* contacts (e.g., labeled as ‘A:B’ or ‘A:G’ for chain A and adjacent chains B and G, respectively). The change in *S* upon inclusion of interface contacts is denoted as Δ*S*. High positive values for Δ*S* suggest that strong selective pressures are maintaining 3D contacts between adjacent subunits. In contrast, values for Δ*S*<0.01 suggest that subunit interactions are not subject to detectable selective constraints. Because SPARC focuses on specific interactions that involve constrained residue sidechains, we add hydrogen atoms to structures and compute the distance between residues based on all the atoms, not just on α- or β-carbons. Specifically, hydrogen bonds are computed based on the distances among donor, acceptor, and hydrogen atoms [63]. SPARC also imposes an ordering on residue interactions, such that closer interactions are ranked higher than those further apart within a cutoff of 4 Å, by default. SPARC computes *S* = -log_10_(*p*) using ICA [4].

**Availability**

SPARC, our other programs, and C++ source code are available at https://www.igs.umaryland.edu/labs/neuwald/software/. All relevant data are within this paper and its supporting information files or at this website or (for very large data sets) from the authors upon request.

## CRediT authorship contribution statement

**Andrew F. Neuwald:** Supervision, Conceptualization, Methodology, Software, Validation, Formal analysis, Investigation, Resources, Data Curation, Visualization, Project administration, Writing – original draft, Funding acquisition. **Hui Yang:** Investigation, Methodology, Resources, Writing – original draft. **B. Tracy Nixon:** Project administration, Supervision, Conceptualiztion, Methodology, Resources, Writing – review & editing, Funding acquisition.

## Declaration of Competing Interest

The authors declare that they have no known competing financial interests or personal relationships that could have appeared to influence the work reported in this paper.
