# Supplementary figures and images for: SPARC: Structural properties associated with residue constraints
Source: Comput Struct Biotechnol J. 2022 Apr 7;20:1702–15. doi: 10.1016/j.csbj.2022.04.005 (PMC9020082; doi:10.1016/j.csbj.2022.04.005)

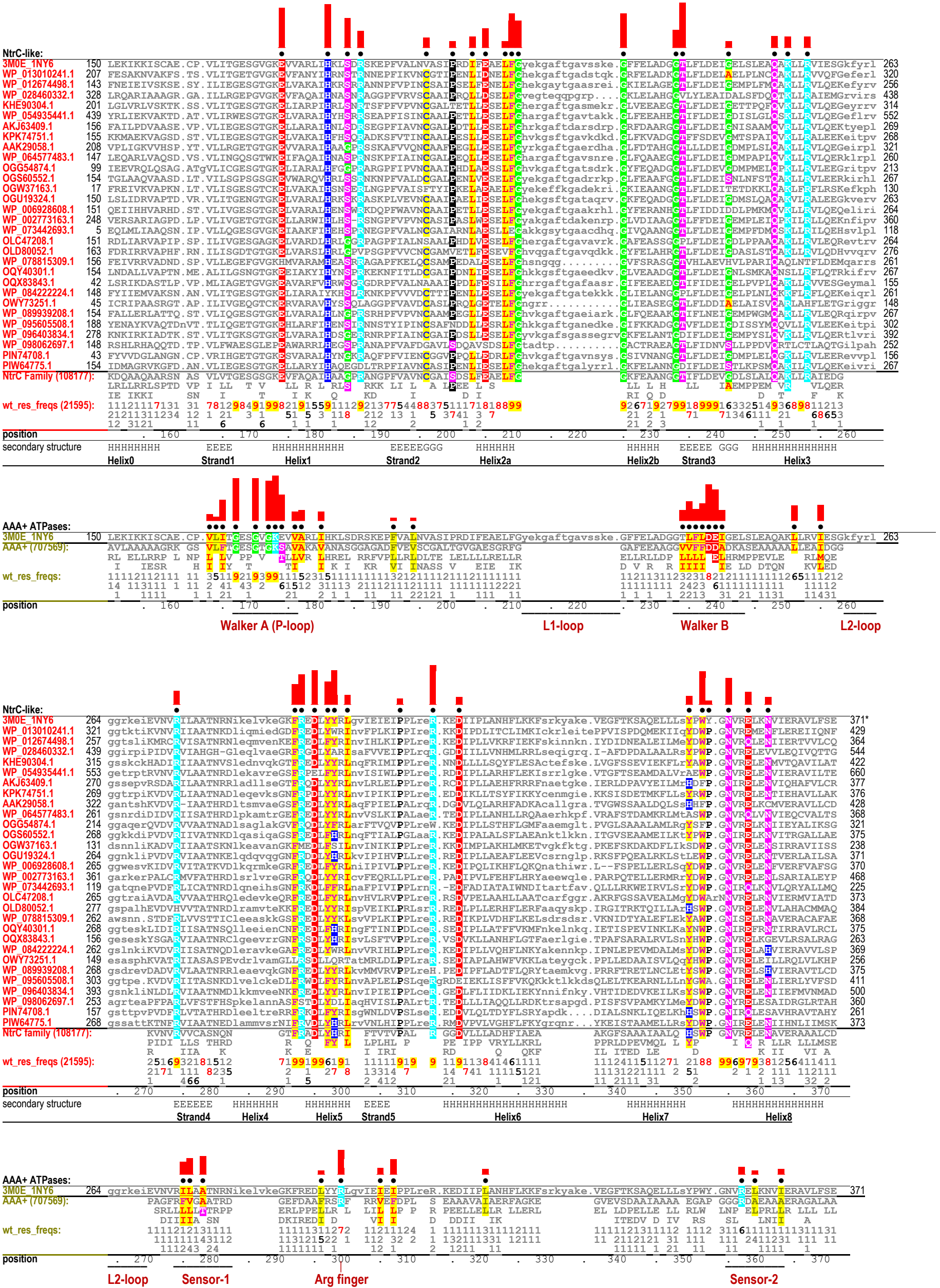

Supplement: Supplementary data 1 [file mmc1.pdf]
