# Supplementary material for: SPARC: Structural properties associated with residue constraints
Source: Comput Struct Biotechnol J. 2022 Apr 7;20:1702–15. doi: 10.1016/j.csbj.2022.04.005 (PMC9020082; doi:10.1016/j.csbj.2022.04.005)
Supplement: Supplementary data 2 [file mmc2.pdf]

Figure S2. BPPS generated contrast alignments showing DnaB family and RecA-like superfamily characteristically conserved residues. Shown is an aligned set of representative bacterial bEBP proteins from distinct phyla that are closely related to the first sequence, DnaB from *E. coli* (pdb_id: 6qem). Aligned residues are highlighted to reveal conserved patterns characteristic of the RecA superfamily, the DnaB-like family and the DnaB subfamily and to indicate amino acid biochemical properties based on the following color code: red font with yellow highlight, non-polar (AVILMWFY) ; blue font with yellow highlight, cysteine (C); red, acidic (DE); cyan, basic (KR); magenta, polar (STNQ); green, glycine (G); blue, histidine (H); black, proline (P). Non-distinguishing positions and nonconserved residues in pattern positions are shown in gray font. The red bars above highlighted columns in the last three alignments quantify the selective pressures imposed on pattern residue positions using a semi-logarithmic scale. Directly below the aligned sequences, the characteristic pattern residues at each position are shown and, directly below these, corresponding frequencies are given in integer tenths. A '7', for example, indicates that the corresponding residue occurs in 70-80% of the foreground sequences. Below the main alignment, pattern residues characteristic of each subgroup are similarly highlighted.

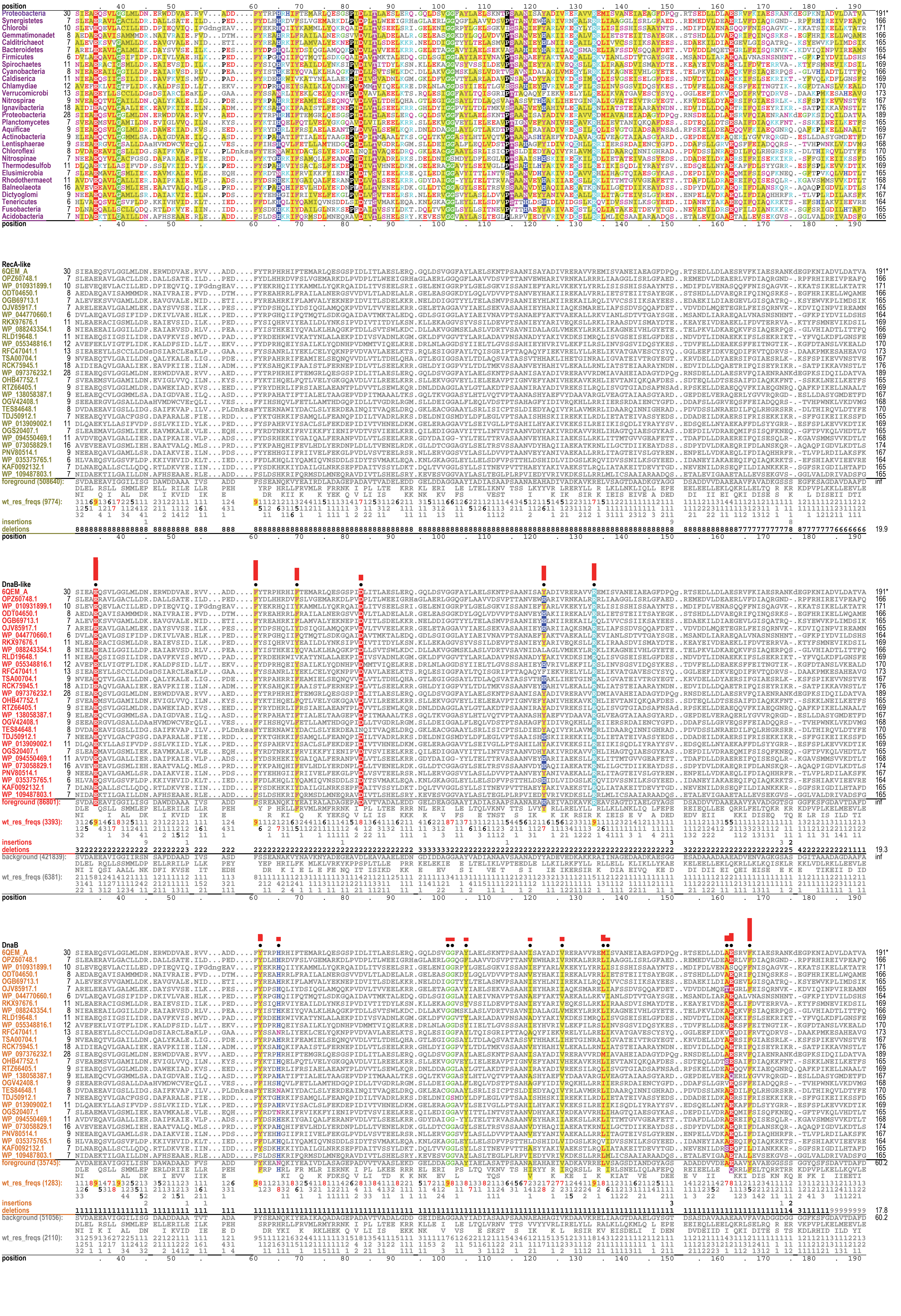

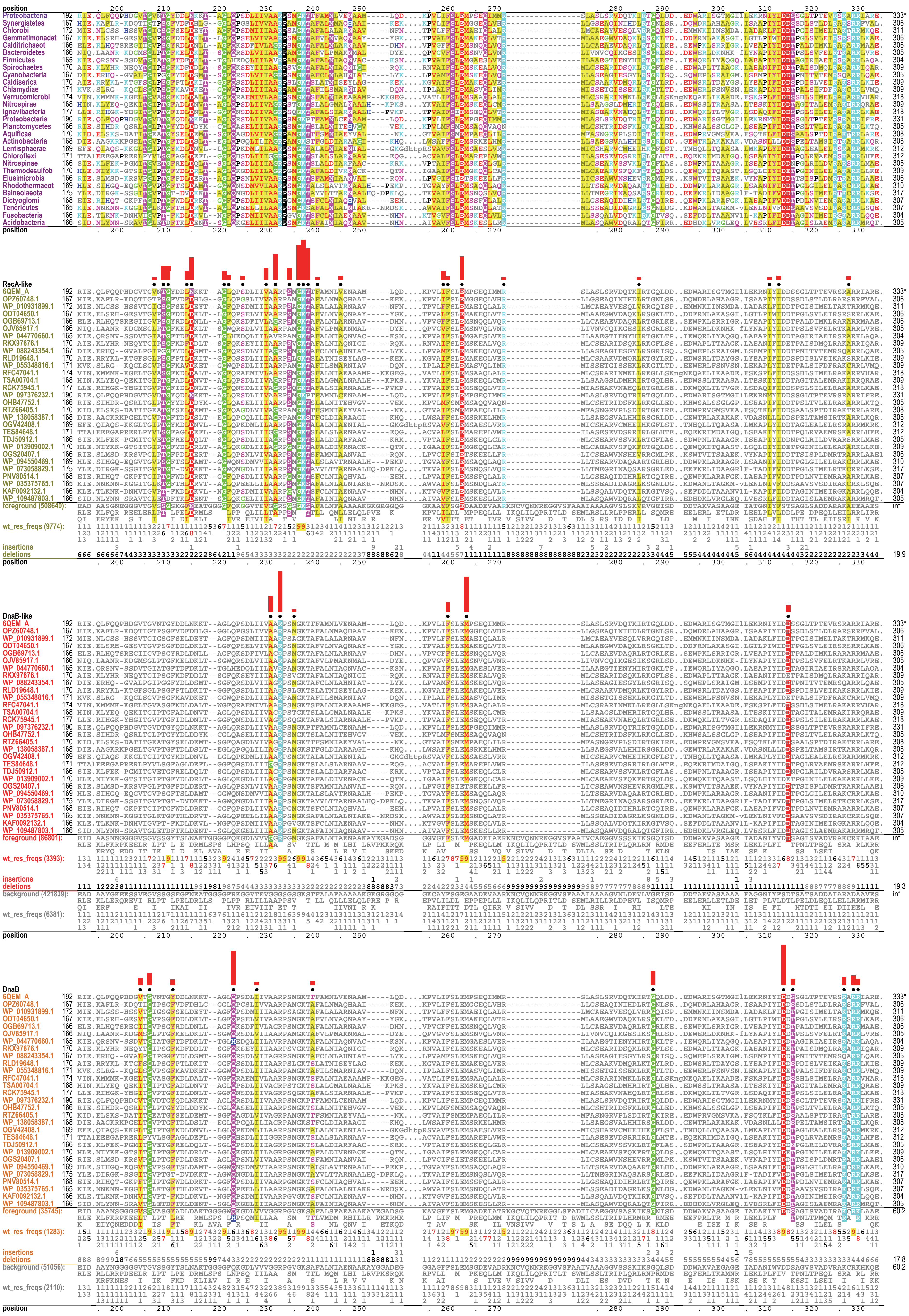
position
RecA-like
DnaB-like
DnaB
6QEM_A

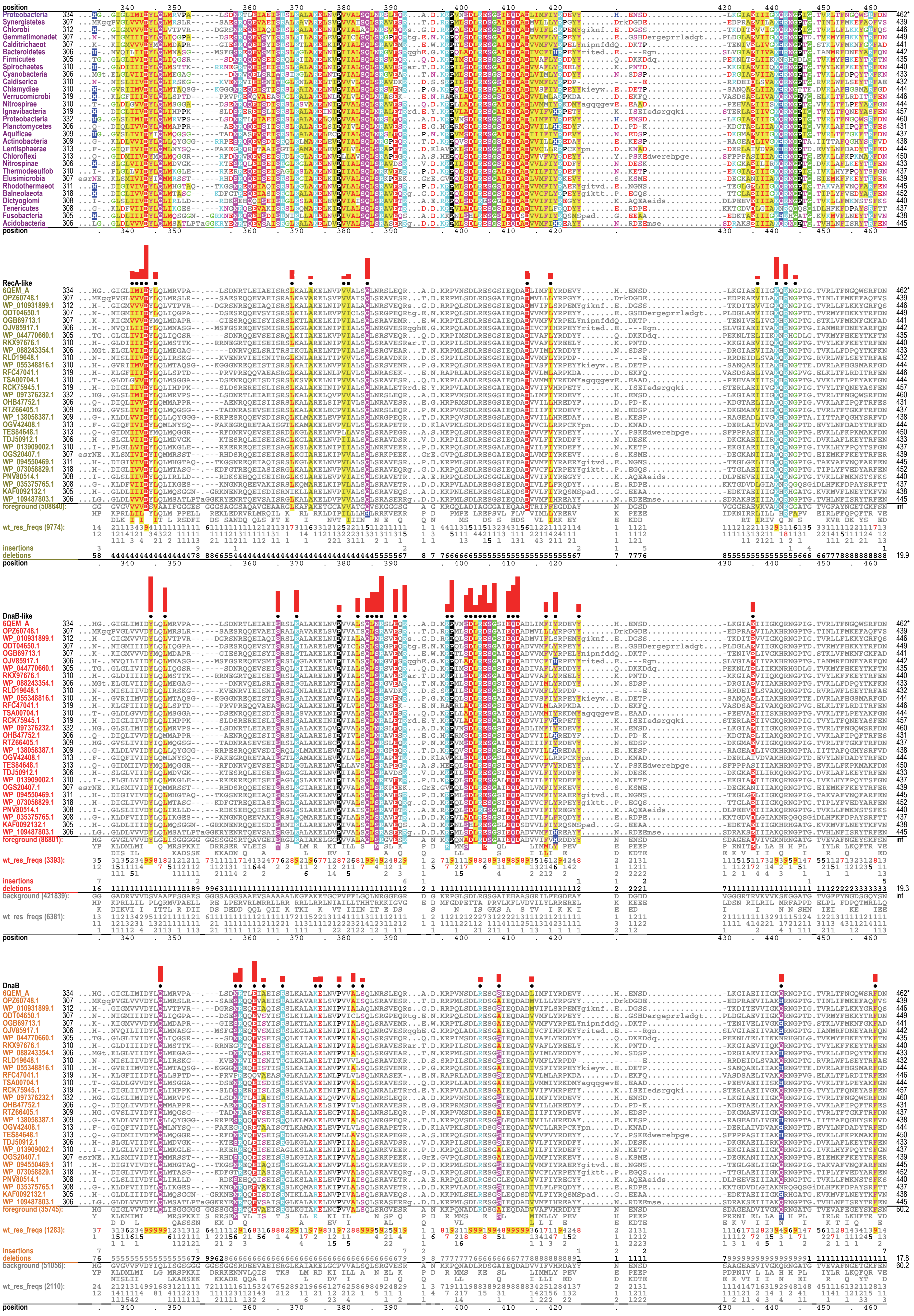

position
RecA-like
DnaB-like
DnaB
